# Combining triptolide with ABT-199 is effective against acute myeloid leukemia through reciprocal regulation of Bcl-2 family proteins and activation of the intrinsic apoptotic pathway

**DOI:** 10.1038/s41419-020-02762-w

**Published:** 2020-07-22

**Authors:** Yuan-Fei Shi, Long Liu, Ling-Li He, Jing Ye, Zhi-Juan Lin, De-Lin Yuan, Man-Man Deng, Zhi-Hong Fang, Bing Z. Carter, Bing Xu

**Affiliations:** 1 Department of Hematology, The First Affiliated Hospital of Xiamen University and Institute of Hematology, Medical College of Xiamen University, Xiamen, Fujian, China; 2 Xiamen Key Laboratory of Diagnosis and Therapy for Hematological Malignancies, Xiamen, Fujian, China; 3 Department of Pediatrics, The Affiliated Zhuzhou Hospital, Xiang Ya Medical College, Central South University, Zhuzhou, Hunan China; 4 Section of Molecular Hematology and Therapy, Department of Leukemia, The University of Texas MD Anderson Cancer Center, Houston, TX USA

**Keywords:** Drug regulation, Drug development

## Abstract

Bcl-2 inhibitors display an effective activity in acute myeloid leukemia (AML), but its clinical efficacy as a monotherapy was limited in part owing to failure to target other antiapoptotic Bcl-2 family proteins, such as Mcl-1. In this context, the combination strategy may be a promising approach to overcome this barrier. Here, we report the preclinical efficacy of a novel strategy combining ABT-199 with triptolide (TPL), a natural product extracted from a traditional Chinese medicine, in AML. Combination treatment exhibited markedly increased cytotoxicity in leukemic cells irrespective of p53 status while largely sparing normal cells of the hematopoietic lineage. Moreover, co-administration of ABT-199 with TPL dramatically suppressed leukemia progression as well as prolonged animal survival in a xenograft AML model. The potentiated effect of ABT-199 and TPL against AML was associated with activation of the mitochondrum-related intrinsic apoptotic pathway through a mechanism reciprocally modulating Bcl-2 family proteins. In this case, TPL not only downregulated Mcl-1 but also upregulated proapoptotic BH3-only proteins, thereby overcoming the resistance toward ABT-199. Conversely, ABT-199 abrogated Bcl-2-mediated cytoprotection against TPL. Together, these findings suggest that the regimen combining TPL and ABT-199 might be active against AML by inducing robust apoptosis through reciprocal regulation of anti- and proapoptotic Bcl-2 family proteins, therefore providing a strong rationale for the clinical investigation of this combination regimen for the treatment of AML.

## Introduction

Targeted therapies for the treatment of acute myeloid leukemia (AML) have been emerging as a promising strategy to overcome chemo-resistance^1^. Among numerous potential targets, Bcl-2, an antiapoptotic protein, has been well characterized as one of the most attractive targets involved in chemo-resistance^2,3^. However, the clinical outcome of AML patients who received the Bcl-2 inhibitor ABT-199 (also known as venetoclax), which has been approved by the FDA for the treatment of chronic lymphocytic leukemia, as monotherapy was discouraging^4^. Therefore, rational combination approaches may be needed to improve the efficacy of ABT-199 in AML. In this context, when combined with azacitidine, ABT-199 displays superior antileukemia activity in AML patients^4–6^.

Mechanistically, ABT-199 acts as a BH3-mimetic, which is highly selective toward Bcl-2^7^. However, Bcl-2 inhibition could incur the upregulation of Mcl-1, another antiapoptotic protein of the Bcl-2 family, thereby resulting in the resistance to ABT-199 in AML^8,9^. In this case, although ABT-199 antagonizes the antiapoptotic function of Bcl-2 by releasing BH3-only proteins (e.g., Bim), it does not increase the levels of these proteins, another major factor that determines their proapoptotic effects^10^. For example, upregulation of BH3 proteins by DNA damage or stress could bind to and activate either or both of the proapoptotic multi-domain protein Bcl-2-associated X protein (BAX) and Bcl-2 antagonist/killer (BAK), triggering apoptosis via mitochondrial outer membrane permeabilization^11^. As the proapoptotic proteins including BH3-only proteins as well as multi-domain proteins can bind to and be neutralized by multiple antiapoptotic proteins, e.g., Bcl-2, Bcl-xL, Mcl-1, either upregulation of other antiapoptotic proteins (particularly, Mcl-1) or insufficient amount of BH3-only proteins, or both could confer resistance to highly selective Bcl-2 inhibitors like ABT-199 and therefore contribute to the unfavorable clinical outcome of ABT-199 as single agent. Rationally, a combination strategy encompassing both aspects, i.e., disability or downregulation of antiapoptotic proteins (e.g., Bcl-2, Mcl-1) and unleashing or upregulation of proapoptotic BH3-only proteins, may thus overcome such resistance and improve the clinical outcome of Bcl-2 inhibitors like ABT-199.

As reported previously, Mcl-1 was upregulated in chemo-resistant AML cells, an event resulting in intrinsic resistance to ABT-199^11^. One approach to overcome this resistance is to combine ABT-199 with an Mcl-1 inhibitor, which indeed enhances inhibition of AML cell growth^12^. However, similar to ABT-199, the Mcl-1 inhibitor has no effect on the levels of BH3-only proteins. Interestingly, it has been reported that the MDM2 inhibitor RG, which activates p53, in combination with ABT-199 exhibits superior antileukemic efficacy and overcome ABT-199 resistance through simultaneous antagonism of Mcl-1 and upregulation of BH3-only proteins^13^. But this combination strategy was effective only toward p53 wild-type AML cells, but not mutant or null cells. In this context, triptolide (TPL) has been demonstrated to execute its antitumor activity by inducing apoptosis via both p53-dependent and -independent mechanisms^14–16^. Mechanistically, this agent acts to increase the levels of BH3-only proteins (e.g., BIM and BID) as well as to downregulate expression of Mcl-1 in various malignant cells^17,18^. Considering its properties of not only suppressing Mcl-1 but also upregulate BH3-only proteins, TPL with a broader antileukemia spectrum might thus represent an optimal agent to potentiate antitumor activity of ABT-199 in AML. Here, we report that TPL interacts with ABT-199 to induce apoptosis and overcome ABT-199-resistance through reciprocal regulation of anti- and proapoptotic Bcl-2 family proteins in AML.

## Materials and methods

### Chemicals and reagents

TPL (formula: C20H24O6, MW: 360.40) and ABT-199 were purchased from Sigma-Aldrich (St. Louis, Missouri, USA) and AbbVie (Chicago, IL, USA), respectively. All compounds were prepared as 10 mm stock solution in 100% dimethyl sulfoxide (Sigma-Aldrich, St Louis, Missouri, USA) and stored at −20 °C or −80 °C. They were diluted to the required concentrations with culture medium immediately before the experiments.

### Cell culture and patient samples

MV4–11 and MOLM-13 (TP53 wild type, FLT3-ITD positive) and U937, THP-1, and KG-1a (TP53 mutated or null), as well as MV4–11-luciferase-GEP cells were kindly provided by professor PT Liu (Wellcome Trust Sanger Institute, UK). Cells were cultured in RPMI-1640 medium or Iscove’s modified Dulbecco’s medium (HyClone, Thermo Scientific, MA, USA) supplemented with 10% fetal bovine serum (FBS; Gibco, Life Technologies, NY, USA) at 37 °C in a humidified CO_2_ incubator. Bone marrow samples were obtained with the informed consent from healthy donors of hematopoietic stem cell transplantation (*n* = 10) and AML patients (*n* = 40) at Department of Hematology, the First Affiliated Hospital of Xiamen University (Xiamen, China). Bone marrow mononuclear cells (BMMC) were isolated by density gradient centrifugation using Lymphoprep (BD, Franklin Lakes, NJ, USA) and cultured in RPMI-1640 medium supplemented with 10% FBS. This study was approved by the Ethics Committee of the First Affiliated Hospital of Xiamen University and conducted in agreement with the guidelines of the Declaration of Helsinki and.

### Cell proliferation assay

The cytotoxic effect of TPL and ABT-199 toward AML cells was determined by Cell Counting Kit-8 (CCK-8) assay (Dojindo, Kumamoto, Japan). In brief, cells (2 × 10^4^ cells/well) were seeded in 96-well plates containing 100 μl growth medium and treated with designated doses of TPL or ABT-199 alone or in combination for 24 or 48 h. CCK-8 reagents (10 μl/well) were then added and incubated for additional 1–3 h, after which the absorbance at 450 nm was detected by a microplate reader (ELx800; BioTek Instruments Inc., Winooski, VT, USA). The results were obtained from at least three independent experiments and expressed as a mean for percentage of living cells by comparing to untreated control in the same experiment. The inhibitory rate was calculated using the following formula: inhibition rate (%) = (1 − absorbance of experimental group/absorbance of control group) × 100%.

### Flow cytometric assay for determining apoptosis and mitochondrial membrane potential

To assess apoptosis, cells were treated with TPL and ABT-199 alone or in combination for 24 and 48 h, followed by staining with Annexin-V-FITC/PI (eBioscience, San Diego, California, USA) for 15 min at room temperature in the dark according to the manufacturer’s instructions. Cells were then analyzed by NovoCyte flow cytometry with NovoExpress software (ACEA Biosciences, Inc., USA) to determine the percentage of Annexin-V positive (apoptotic) cells. Loss of the mitochondrial membrane potential (Δψm) was measured using JC-1 Fluorescent Probe Kit (Beyotime Company, Shanghai, China) by following the manufacturer’s instructions as described previously^15^. To assess the interaction between TPL and ABT-199, two separate approaches (i.e., CompuSyn and CalcuSyn) were used to calculate the combination index (CI). CI < 1.0 indicates synergism, while CI = 1.0 and CI > 1.0 indicate an additive or antagonistic effect, respectively.

### Western blot analysis

After treatment, a total 5 × 10^6^/ml cells per condition were lysed and the protein concentration was determined using BCA Protein Assay Kit (Pierce, Rockford, IL, USA). Whole cell lysates (50 μg for each sample) were electrophoresed in 10% sodium dodecyl sulphate polyacrylamide gel electrophoresis and then transferred to polyvinylidene difluoride membrane (Millipore, Billerica, MA, USA). The membranes were incubated with primary antibodies (1:1000 in 5% bovine serum albumin tris-buffered saline with polysorbate 20; TBS-T) overnight at 4 °C, followed by horseradish peroxidase-conjugated secondary antibodies (Cell Signaling Technology; 1:10,000 in 5% fat-free milk TBS-T) for 1.5 h at room temperature. The primary antibodies against Bcl-2 (CA4223S), Mcl-1 (CA2538S), caspase 3 (CA9662S), caspase 9 (CA5340S), PARP (CA9532S), BIM (CA2933S), PUMA (CA3741S), BID (CA7382S), and β-actin (CA4970S) were purchased from Cell Signaling Technology (Danvers, MA, USA). The blots were probed with anti-β-actin antibody (rabbit mAb 1:1000, Cell Signaling Technology) to ensure equal loading and transfer. The proteins were visualized using ECL Western Blotting Detection Kit (Gene-Flow, Staffordshire, UK).

### Animal study

The animal study was approved by the First Affiliated Hospital of Xiamen University Animal Care and Use Committee. Two xenograft models were employed in this study. For the model #1, 5~6 week-old female BALB/C nude mice (18–20 g; Beijing HFK bioscience Co. Ltd.) were injected subcutaneously with 5.8 × 10^6^ MOLM-13 cells at the right flank of mice. Once the tumor volume reached to ~75 mm^3^, mice were randomly assigned to four groups (*n* = 5/group), including control, TPL, ABT-199, and combination, and treated for 2 consecutive weeks with vehicle (0.2% methyl cellulose and 0.1% Tween-80 in PBS), TPL (0.5 mg/kg/day, intraperitoneal injection), ABT-199 (50 mg/kg/day, oral gavage), or combination of TPL and ABT-199, respectively. During treatment, mice were monitored and weighed daily for toxicity. After 2 weeks of treatment, tumor volume (V) was measured daily by caliper and calculated using the equation: *V* = (*L* × *W*^2^)/2, where *L* and *W* represent the length and width of tumor, respectively.

For the model #2, six-week-old male NOD-SCID IL-2Rγ null (NSG) mice (BEIJING IDMO Co. Ltd, China) were injected intravenously with 4 × 10^6^ MV4–11-luciferase-GFP cells. After engraftment was confirmed, mice were randomly assigned to four groups (*n* = 7/group) and treated daily with vehicle, TPL (0.5 mg/kg/day, intraperitoneal injection), ABT-199 (50 mg/kg/day, oral gavage), or their combination. The tumor burden was monitored by bioluminescent imaging at designated time points. In brief, mice were anesthetized and injected intraperitoneally with the firefly luciferase substrate d-luciferin (Gold Biotechnology, St. Louis, MO, USA) and then imaged using IVIS-200 Imaging System (PerkinElmer, Waltham, MA, USA). Five mice in each group were used for the analysis of animal survival. Other two mice in each group were killed at day 21 for control group, day 28 for ABT-199 or TPL group, and day 35 for combinatory group. Spleens were removed and subjected to immunohistochemical staining with human CD45 antibody or western blot analysis on whole tissue homogenate.

### Statistical analysis

The value was expressed as the mean ± standard deviation (S.D.) for at least three independent experiments. Two groups were compared using the Student *t* test. Multiple-group comparisons were conducted using the one-way analysis of variance followed by the Bonferroni post hoc test. Survival was estimated using the Kaplan–Meier analysis and analyzed with the log-rank test. *P* < 0.05 was considered statistically significant. All statistical analyses were performed using SPSS 20.0 software (La Jolla, CA).

## Results

### Co-treatment with TPL and ABT-199 markedly induces apoptosis in AML cells, irrespective of p53 status

**Fig. 1:**
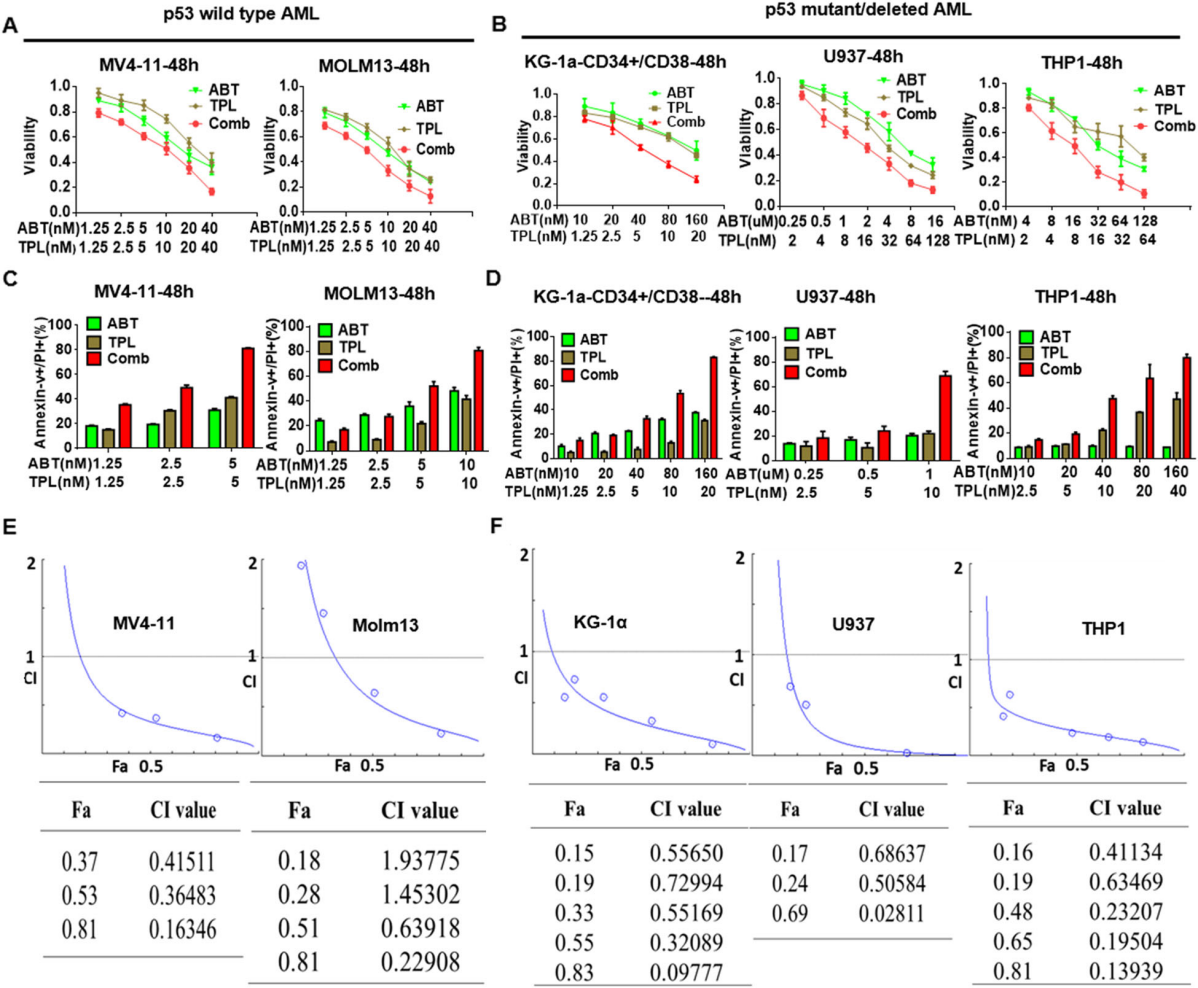
Combination of TPL and ABT-199 induces apoptosis in p53 wild type and mutant/deleted AML cells. **a**, **b** Inhibition on proliferation of both p53 wild type (MV4–11 and MOLM-13) and mutant/null AML cells (KG-1a, U937, and THP-1) were exposed to the indicated concentrations of TPL ± ABT-199 for 48 h, after which the CCK-8 assay was performed to monitor cell viability. **c**, **d** Alternatively, cells were treated with the indicated concentrations of TPL ± ABT-199 for 48 h, after which the percentage of Annexin-V+apoptotic cells was determined by flow cytometry after Annexin-V and PI double staining. **e**, **f** The combination index (CI) was calculated based on apoptosis using the CompuSyn and CalcuSyn software to evaluate the interaction between TPL and ABT-199 in both p53 wild type and mutant/null AML cell lines (CI < 1.0 = 1.0, and >1.0, indicating synergistic, additive, and antagonistic effect, respectively). ABT, ABT-199; Comb: combination of TPL and ABT-199.

First, we examined whether the combination of TPL and ABT-199 would have an interactive effect on AML cells. To this end, various leukemia cell lines (including p53 wild type and mutation/null) were treated with indicated concentrations of TPL and ABT-199, after which cell viability was analyzed using CCK-8 assay. As shown in Fig. 1a, b, treatment with TPL and ABT-199 alone significantly inhibited cell growth in a dose-dependent manner in both p53 wild type (MV4–11 and MOLM-13, both carrying FLT3-ITD, a genetic marker for poor prognosis; Fig. 1a) and mutant or null cells (U937, THP-1, and KG-1α; Fig. 1b). Notably, co-administration of TPL and ABT-199 led to markedly enhanced growth inhibition in all of these AML cell lines (Fig. 1a, b). To verify whether the cytotoxicity of TPL and ABT-199 was associated with induction of apoptosis, the percentage of apoptotic cells was determined by Annexin-V/PI dual staining. As shown in Fig. 1c, d, each single-agent treatment resulted in a modest increase in apoptosis, whereas the percentage of apoptotic cells was dramatically increased after co-exposure to TPL and ABT-199. CI values were further determined using the Calcusyn and CompuSyn software, respectively. CI < 1.0 for virtually all conditions (except two conditions for Molm13) indicates synergism between TPL and ABT-199 in both p53 wild type (Fig. 1e and Supplementary Fig. 1A, B) and mutant/null AML cell lines (Fig. 1f and Supplementary Fig. 1C–E). These results were confirmed by another analysis using the Bliss Independence model (Supplemental Table S1). Together, these findings suggest that the combination of TPL and ABT-199 might markedly reduced cell viability and markedly induce apoptosis in AML cells regardless of their p53 status.

### Combined treatment with TPL and ABT-199 results in an enhanced cytotoxicity against primary AML blasts, whereas largely sparing normal BMMCs

**Fig. 2:**
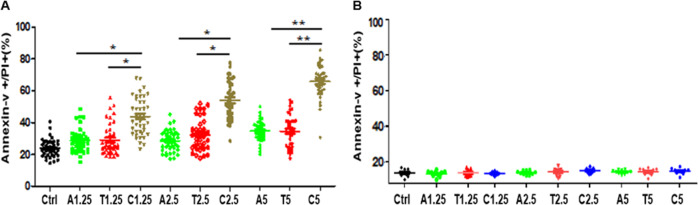
Combination of TPL and ABT-199 selectively targets primary AML blasts, while sparing normal BMMCs. **a**, **b** Primary blasts and bone marrow mononuclear cells (BMMC) were isolated from 40 patients with AML and 10 healthy donors, respectively. Cells were exposed to the indicated concentrations (nm) of TPL (T) or ABT-199 (A) alone or in combination **c** for 48 h, after which the percentage of Annexin-V + apoptotic cells was determined by flow cytometry after Annexin-V and PI double staining. *P < 0.05, **P < 0.01.

The antileukemic effect of TPL and ABT-199 alone or in combination was further investigated ex vivo in the primary blasts isolated from 40 AML patients and BMMCs from 10 healthy donors. Clinical characteristics of the patients were summarized in Table 1. Consistent with the results obtained from AML cell lines, co-treatment of primary AML blasts with TPL and ABT-199 at the concentrations ranging from 1.25 to 5 nmol/L resulted in significant increased in the percentage of apoptotic cells when compared with each agent alone (Fig. 2a). In sharp contrast, combined treatment with TPL and ABT-199 at the same dose range had almost no cytotoxicity toward BMMCs obtained from healthy donors (Fig. 2b). Together, these results suggest that the combination of TPL with ABT-199 might be preferably targeted AML blasts while potentially sparing normal cells of the hematopoietic lineage.

**Table 1:** Clinical characteristics of patients enrolled in this study.

Patient	Gender	Age (yr)	WBC(10^9/L)	Cytogenetic risk
1	M	27	7.25	Low
2	M	62	44.43	Intermediate
3	M	65	49.29	Intermediate
4	M	66	48.34	Intermediate
5	F	64	1.60	Intermediate
6	M	53	53.25	Intermediate
7	M	44	0.270	Low
8	M	61	97.34	Low
9	M	60	5.210	Intermediate
10	M	38	19.82	Intermediate
11	M	58	36.48	Low
12	M	44	3.570	Intermediate
13	M	32	324.4	Low
14	F	57	8.540	Intermediate
15	M	65	35.32	Intermediate
16	M	29	22.23	Intermediate
17	M	65	7.380	High
18	M	63	11.64	Intermediate
19	F	34	2.610	Intermediate
20	M	66	2.74	Intermediate
21	F	28	1.56	Low
22	F	9	581.8	Intermediate
23	F	7	13.53	Intermediate
24	M	29	22.23	Intermediate
25	F	11	32.4	Intermediate
26	F	34	2.61	Intermediate
27	F	56	8.27	High
28	F	7	342	Intermediate
29	F	9	532	Intermediate
30	F	13	271	Intermediate
31	M	28	32.23	Intermediate
32	F	61	87.34	Low
33	M	68	55.32	Intermediate
34	M	66	56.20	Intermediate
35	M	59	13.2	Intermediate
36	M	73	21.64	Intermediate
37	F	34	15.3	Intermediate
38	F	7	13.53	Intermediate
39	F	56	28.27	High
40	M	27	7.25	Low

### The regimen combining TPL and ABT-199 displays antileukemic activity in murine xenograft models

**Fig. 3:**
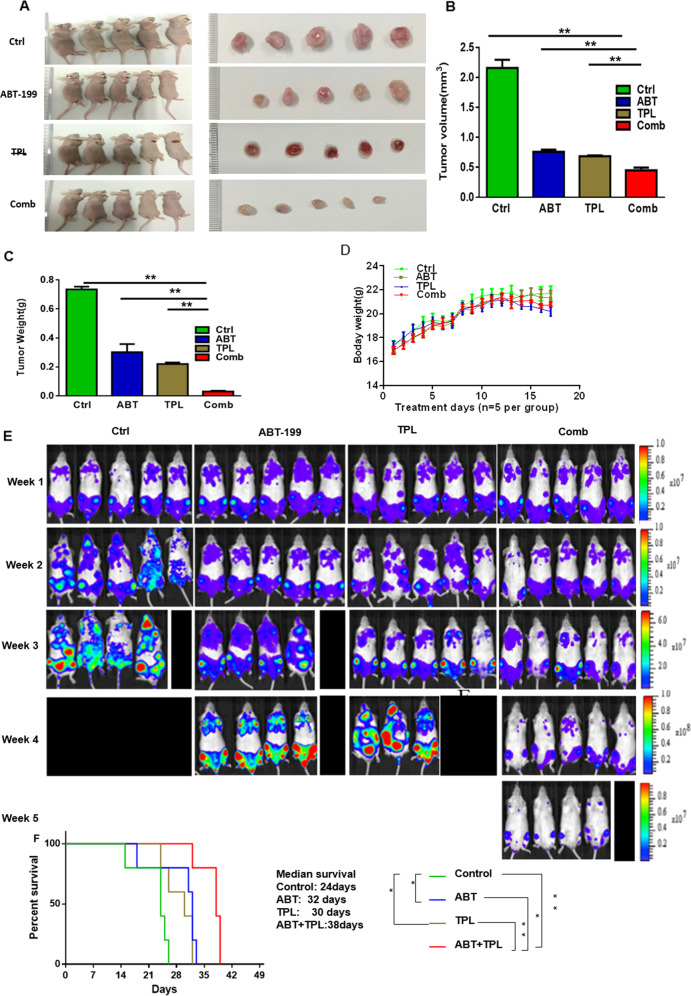
Combination of TPL and ABT-199 displays a superior antileukemic activity *in vivo*. **a**–**d** BALB/C nude mice were injected subcutaneously with 5.8 × 10^6^ MOLM-13 cells at the right flank. Once the tumor volume reached to ~75 mm^3^, mice were randomly assigned to four groups (*n* = 5/group) including control (ctrl), TPL, ABT-199, and combination (combo), and treated for 2 consecutive weeks with vehicle (0.2% methyl cellulose and 0.1% Tween-80 in PBS), TPL (0.5 mg/kg/day, intraperitoneal injection), ABT-199 (50 mg/kg/day, oral gavage), or combination of TPL and ABT-199, respectively. At the end of the study, images of mice and removed tumors were captured **a**, and tumor volume **b** and weight **c** were measured. During treatment, body weight of mice was monitored daily **d**. Data represent the mean ± SD. **e**, **f** NOD-SCID IL-2Rγ null mice were injected intravenously with 4 × 10^6^ MV4–11-luciferase-GFP cells. After engraftment was confirmed, mice were randomly assigned to four groups (*n* = 7/group) and treated daily with vehicle (ctrl), TPL (0.5 mg/kg/day, intraperitoneal injection), ABT-199 (50 mg/kg/day, oral gavage) or their combination (Combo). The tumor burden was monitored by bioluminescent imaging at designated time points **e**. Animal survival was analyzed using the Kaplan–Meier survival curve **f**. *P < 0.05, **P < 0.01.

We next evaluated the activity and toxicity of the regimen combining TPL and ABT-199 in two xenograft models bearing either MOLM-13 or MV4–11 cells, both of which carry FLT-ITD, representing an AML subset with poor clinical outcomes. The first model established by subcutaneously inoculating MOLM-13 cell line into flank of mice were treated with vehicle, TPL, ABT-199, or their combination, respectively. Compared with each single agent, combined treatment resulted in a marked inhibition of tumor growth (Fig. 3a), manifested by a significant reduction in tumor volume and weight (Fig. 3b,c). Of note, this combination regimen was well tolerated, without significant loss of body weight (Fig. 3d). Moreover, toxicity of TPL was further examined in non-immunocompromised mice. Neither blood cell account nor liver function was affected when compared with control group (Supplementary Fig. 2), supporting a notion that the dose of TPL used in this study is tolerable.

To further investigate whether the combination of TPL and ABT-199 would confer a survival benefit, the second xenograft model was established by intravenously injecting with GFP/luciferase-labeled MV4–11 cells. Mice were then treated with vehicle, TPL (0.5 mg/kg/day), ABT-199 (50 mg/kg/day) or their combination, respectively. As shown in Fig. 3e, TPL or ABT-199 administrated individually resulted in a moderate reduction in tumor burden, whereas combined treatment remarkably reduced tumor burden. Consistently, although mice received TPL and ABT-199 as single agent displayed longer survival than those only received vehicle, combined treatment led to markedly prolonged survival, compared with each agent alone, with median survival time of 38 days for the combination group vs 32 days for ABT-199 or 30 days for TPL monotherapy group (Fig. 3f, *p* < 0.05 for each case).

### Antileukemic effect of ABT-199 and TPL is associated with activation of the intrinsic apoptotic pathway

**Fig. 4:**
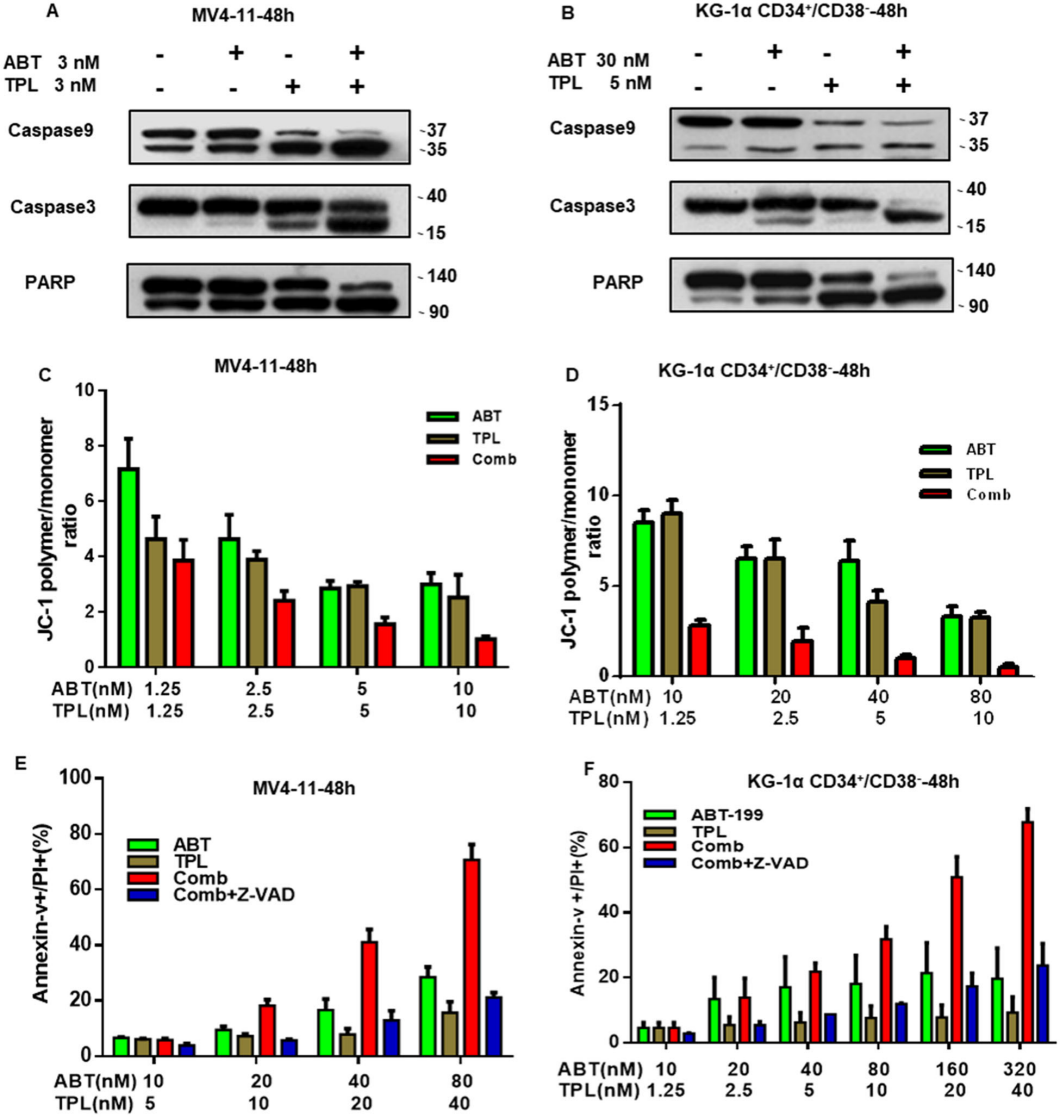
The interaction between TPL and ABT-199 is associated with the mitochondrial intrinsic apoptosis. **a**, **b** MV4–11 and KG-1α cells were exposed to the indicated concentrations of TPL ± ABT-199 for 48 h, after which western blot analysis was performed to monitor cleavage caspase 3, caspase 9, and PARP. **c**, **d** Alternatively, cells were treated with the indicated concentrations of TPL ± ABT-199 for 48 h, after which flow cytometry was performed to assess depolarization of mitochondrial membrane after JC-1 staining. **e**, **f** MV4–11 and KG-1α cells were incubated for 24 h with the indicated concentrations of TPL ± ABT-199 in the absence or presence of the pan-caspase inhibitor Z-VAD-fmk, after which the percentage of apoptotic cells was determined by flow cytometry after Annexin-V and PI staining.

To examine whether cytotoxicity of the TPL+ABT-199 combination is associated with activation of the intrinsic apoptotic pathway, cleavage of caspases and PARP, markers of apoptosis were first monitored. As shown in Fig. 4a, b, treatment with ABT-199 and TPL in combination markedly increased cleavage of caspase 9, caspase 3 and PARP, compared with each agent administrated individually in both p53 wild type MV4–11 (Fig. 4a) and p53 mutant KG-1α cells (Fig. 4b). Flow cytometry was then performed to assess depolarization of mitochondrial membrane. Consistent to induction of apoptosis, co-treatment with TPL and ABT-199 resulted in a marked loss of mitochondrial membrane enhanced in a dose-dependent manner in MV4–11 (Fig. 4c) and KG-1α cells (Fig. 4d), suggesting activation of the mitochondrum-related intrinsic apoptotic pathway. Last, Z-VAD-fmk, a pan-caspase inhibitor, was used to rescue apoptosis induced by the combination treatment. Indeed, Z-VAD-fmk significantly prevent leukemic cells from apoptosis induced by the combination of TPL and ABT-199 in both MV4–11 (Fig. 4e) and KG-1α (Fig. 4f). Together, these results suggest that the antileukemic effect of ABT-199 and TPL in combination might primarily stem from activation of the intrinsic apoptotic pathway.

### Reciprocal regulation of Bcl-2 family proteins contributes to activity of TPL and ABT-199 against AML cells

**Fig. 5:**
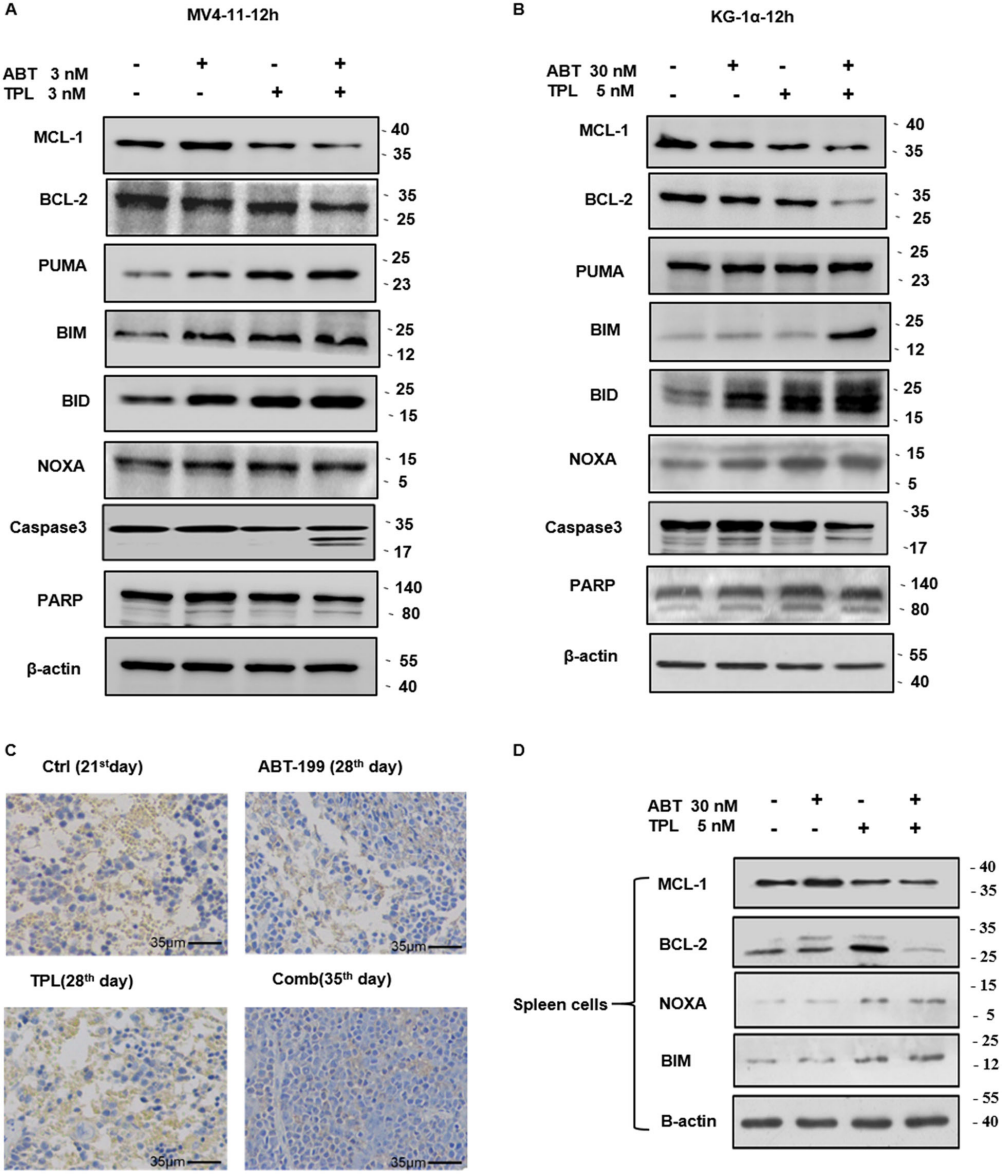
Combination of TPL and ABT-199 reciprocally regulates Bcl-2 family proteins. **a**, **b** MV4–11 and KG-1α cells were exposed to the indicated concentrations of TPL ± ABT-199 for 12 h, after which western blot analysis was performed to monitor expression of proapoptotic BH3-only proteins (PUMA, NOXA, BID, BIM) and antiapoptotic proteins (Bcl-2 and Mcl-1), as well as cleavage of caspase 3 and PARP. **c**, **d** Animal study was performed as described in Fig. 3e, f, after which spleen was removed at the indicated time points (Ctrl, day 21; TPL and ABT-199 alone, day 28; combination, day 35) and subjected to immunohistochemical staining (IHC) for human CD45 **c** (Scale bar, 35 μm) and western blot analysis for BH3-only proteins (NOXA, BIM), Bcl-2, and Mcl-1 on spleen homogenate.

Finally, the mechanism of action of the TPL+ABT-199 combination was investigated. As the effect of TPL and ABT-199 was associated with activation of the mitochondrial apoptotic pathway that is primarily governed by pro- and antiapoptotic proteins of the Bcl-2 family^19^, the expression of Bcl-2 family proteins was monitored at 12 hr after drug treatment, prior to apoptosis (Supplementary Fig. 3). As shown in Fig. 5a, b, whereas ABT-199 barely affect the levels of Bcl-2 and Mcl-1, TPL moderately but clearly reduced the expression of Mcl-1 but not Bcl-2. Notably, combined treatment with TPL and ABT-199 markedly reduced the protein levels of both Bcl-2 and Mcl-1 in MV4–11 (Fig. 5a) and KG-1α cells (Fig. 5b), an event occurred prior to cleavage of caspase 3 and PARP. In parallel, exposure to TPL or ABT-199 alone resulted in a moderate increase in the protein levels of several BH3-only proteins, including PUMA, BIM, BID, and NOXA, co-treatment with two agents dramatically upregulated the expression of these proapoptotic proteins (Fig. 5a, b).

Similar phenomena were observed in infiltrating leukemic cells in spleen of xenograft mice bearing GFP/luciferase-labeled MV4–11 cells. It was noticed that although TPL or ABT-199 as monotherapy displayed a moderate but clear suppressive effect on tumor growth at early time points, neither of them was able to prevent progression of leukemia at late stage (e.g., day 28; Fig. 3e). As shown in Fig. 5c, combined treatment with TPL and ABT-199 markedly reduced the amount of infiltrating leukemia cells in spleen harvested at indicated time points, consistent with the observation that the combination virtually eliminated tumor burden (Fig. 3e). Interestingly, western blot revealed that the protein level of Mcl-1 and Bcl-2 was increased in ABT-199 and TPL group, respectively (Fig. 5d), probably accounting for the insufficiency of either agent alone to prevent disease progression. However, these events were diminished by combination treatment, accompanied by upregulation of proapoptotic BH3-only proteins such as BIM and NOXA (Fig. 5d). Together, these findings argue that TPL and ABT-199 might interact to induce robust apoptosis and overcome resistance to each single drug through disruption of the compensatory responses to these agents alone by simultaneously downregulation of antiapoptotic Bcl-2 family proteins and upregulation of proapoptotic BH3-only proteins in AML cells.

## Discussion

Most targeted therapies focus on upstream nodes of tumor-driven signaling pathways^19^. However, drug resistance is often inevitable especially for the monotherapy of targeted agents due to various compensatory mechanisms via the complex signaling networks^19,20^. In this context, the Bcl-2-selective inhibitor ABT-199 as monotherapy has displayed a limited efficacy due to compensatory upregulation of Mcl-1 and/or incapability to induce BH3-only protein expression^12,21^. Thus, the combination approach is required for overcoming this barrier and thereby potentiate antitumor activity of ABT-199. An ideal partner for such a combination strategy compound should be able to not only enhance proapoptotic effect of ABT-199 by up-regulating BH3-only proteins but also overcome its resistance due to Mcl-1 upregulation. In this study, we found that TPL interacts ABT-199 to markedly induce apoptosis in AML cells irrespective of p53 status. We also noticed that in contrast to transient inhibitory effect of TPL or ABT-199 monotherapy, the regimen combining these two agents displays persistent antileukemic activity in vivo, while is well tolerated.

To overcome Mcl-1-mediated resistance of ABT-199 in AML cells, Mcl-1 inhibitors have been used to reverse this resistance. Indeed, combination of an Mcl-1 inhibitor and ABT-199 did enhance the inhibition of leukemia growth. However, this strategy might be further optimized, because both ABT-199 and Mcl-1 inhibitors are BH3 mimetics that antagonize antiapoptotic proteins of the Bcl-2 family but do not affect the expression of BH3-only proteins in AML cells^21^. In this context, BH3-only proteins are considered as a key factor to initiate the intrinsic apoptotic pathway^10,22^. As ABT-199 or Mcl-1 inhibitors can only release the preexisted BH3-only proteins from binding of neutralization by Bcl-2 or Mcl-1, an ideal candidate partner for combining with ABT-199 would be an agent that not only downregulate or inhibit Mcl-1 but also upregulate BH3-only proteins. For example, it has been demonstrated that a p53 activator in combination with ABT-199 could result in synthetic lethality by both targeting Mcl-1 and up-regulating BH3-only proteins in AML cells^13^. Unfortunately, this combination approach was effective only against p53 wild-type AML, whereas failed to kill AML cells carrying p53 mutation or deletion, a common and poor-prognostic genetic abnormality in this disease. In this study, we found that although AML cells carrying p53 mutation or deletion seemed less sensitive to ABT-199 or TPL than those with wild-type p53, the regimen combining TPL and ABT-199 markedly induced apoptosis and displayed a significantly increased antitumor activity in AML regardless of p53 status. It is noteworthy that the concentrations of ABT-199 and TPL used in this study were comparable to the achievable plasma concentrations in clinical settings^23,24^. In ranges of these concentrations, neither agent alone nor in combination resulted in significant toxicity toward BMMC obtained from healthy donors, suggesting that these dose ranges might be tolerated in vivo. Therefore, the regimen combining TPL with ABT-199 might have a broader antileukemic spectrum in AML carrying both wild type and mutant/deleted p53, with a likelihood of clinical tolerability.

Mechanistically, the marked antileukemic effect of TPL and ABT-199 in AML was associated with activation of the mitochondrum-related intrinsic apoptotic pathway through reciprocal regulation of both anti- and proapoptotic Bcl-2 family proteins. In this case, TPL could not only downregulate Mcl-1 but also upregulate BH3-only proteins, which compensate for the Mcl-1-mediated resistance to ABT-199. Conversely, ABT-199 might rescues Bcl-2-mediated paralysis toward TPL. Together, combination of TPL and ABT-199 might potentially overcome drug resistance to both agents. As a consequence, the regimen combining TPL and ABT-199 displayed a potent and persistent antileukemic efficacy in vitro and in vivo, as well as conferred a superior animal survival in the murine xenograft AML models. However, a possibility that the mechanisms, other than those involving the Bcl-2 family, might also contribute to the interaction between ABT-199 and TPL could not be excluded.

In summary, this study provides strong preclinical evidence supporting that a novel strategy combining TPL and ABT-199 markedly induces apoptosis of AML cells regardless of their p53 status and thereby displays the markedly improved efficacy towards AML in vitro and in vivo. Mechanistically, the interaction between these two agents is associated with reciprocal regulation of anti- and proapoptotic Bcl-2 family proteins, which might thus overcome drug resistance for each single agent administrated as monotherapy. Therefore, this combination regimen warrants further clinical investigation for the treatment of AML patients, particularly those carrying adverse genetic abnormalities such as p53 mutation or deletion.

## Supplementary information

Supplementary Figure 1

Supplementary Figure 2

Supplementary Figure 3

Supplementary Information

Supplementary Table S1
