# Supplementary figures and images for: Combining triptolide with ABT-199 is effective against acute myeloid leukemia through reciprocal regulation of Bcl-2 family proteins and activation of the intrinsic apoptotic pathway
Source: Cell Death Dis. 2020 Jul 22;11(7):555. doi: 10.1038/s41419-020-02762-w (PMC7376040; doi:10.1038/s41419-020-02762-w)

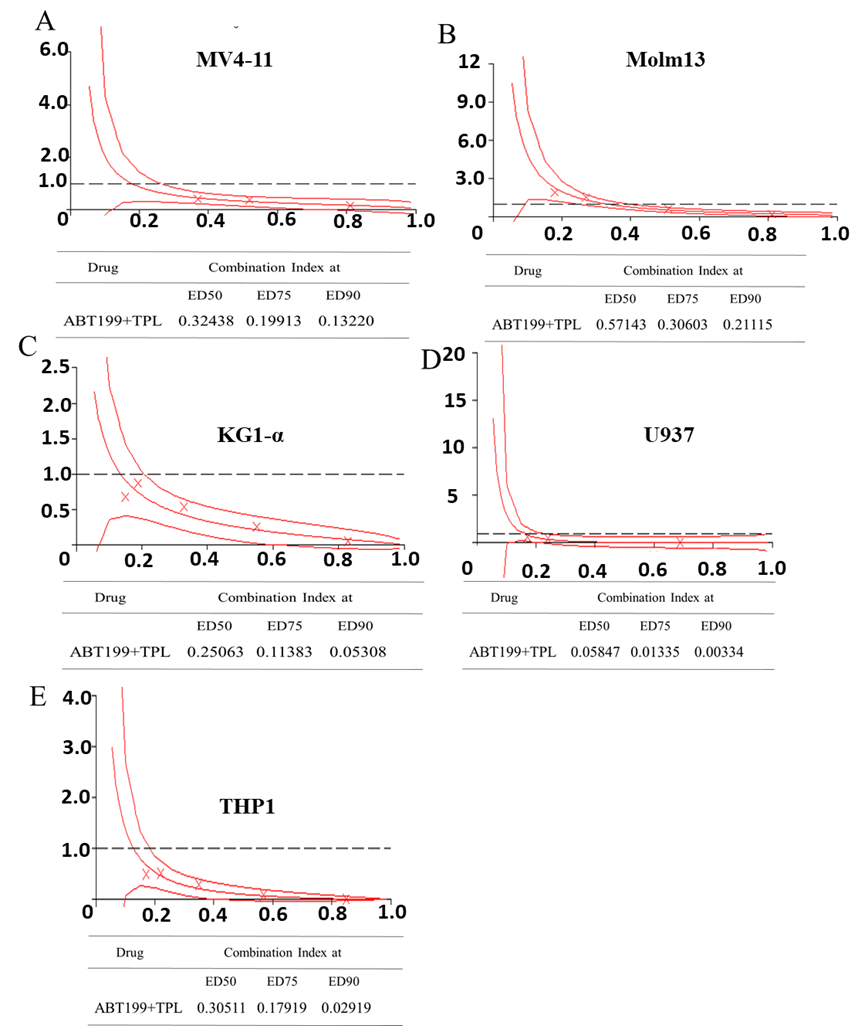

Supplement: Supplementary file 1 — Supplementary Figure 1 [file 41419_2020_2762_MOESM1_ESM.tif]

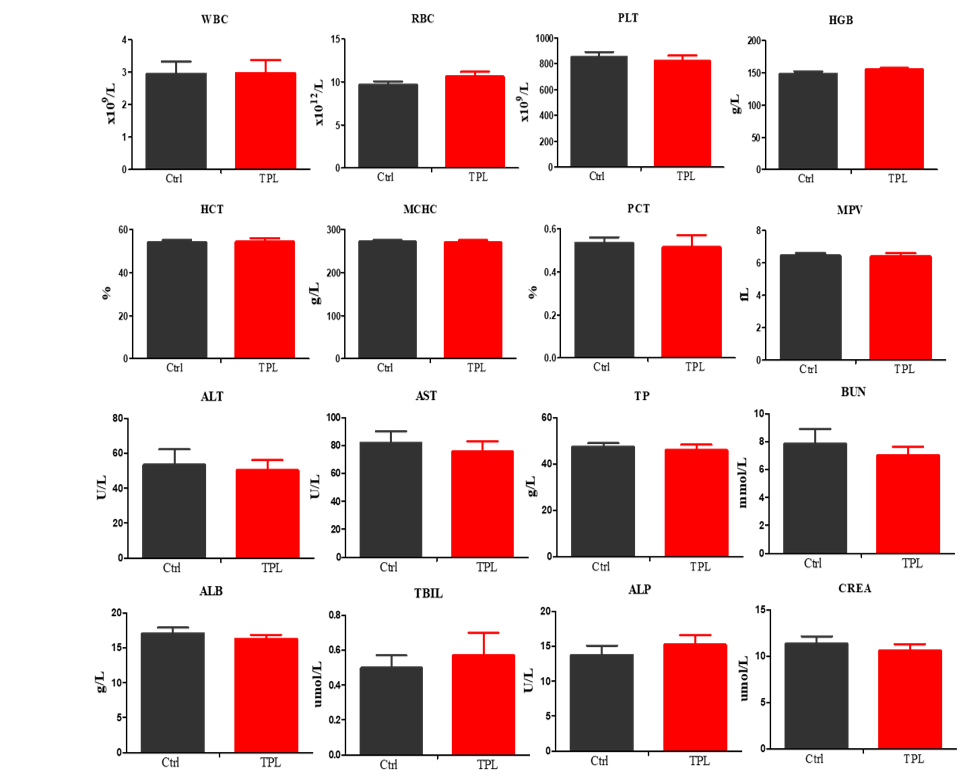

Supplement: Supplementary file 2 — Supplementary Figure 2 [file 41419_2020_2762_MOESM2_ESM.tif]

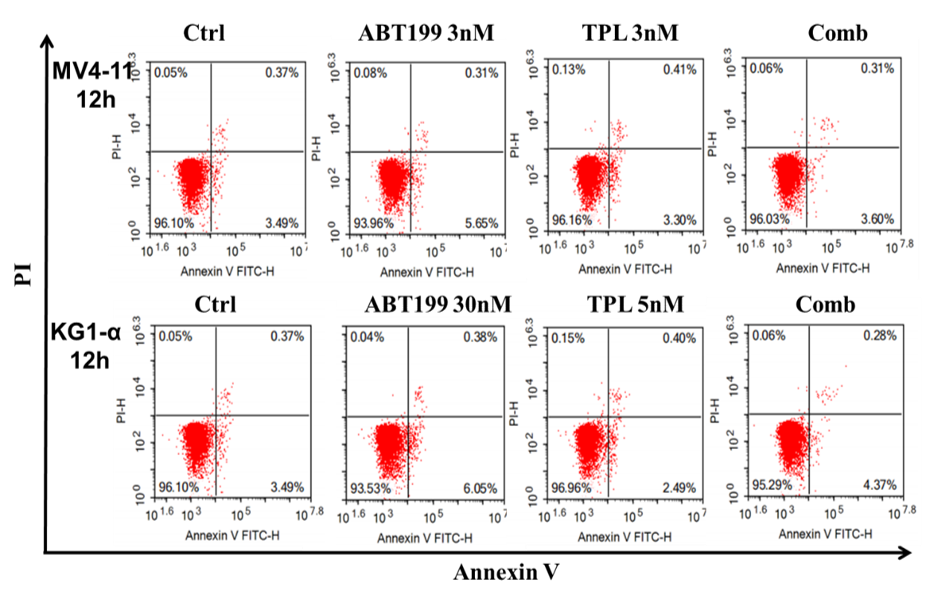

Supplement: Supplementary file 3 — Supplementary Figure 3 [file 41419_2020_2762_MOESM3_ESM.tif]
