# Supplementary material for: Combining triptolide with ABT-199 is effective against acute myeloid leukemia through reciprocal regulation of Bcl-2 family proteins and activation of the intrinsic apoptotic pathway
Source: Cell Death Dis. 2020 Jul 22;11(7):555. doi: 10.1038/s41419-020-02762-w (PMC7376040; doi:10.1038/s41419-020-02762-w)
Supplement: Supplementary file 4 — Supplementary Information [file 41419_2020_2762_MOESM4_ESM.docx]

**Combining Triptolide with ABT-199 is effective against acute myeloid leukemia through reciprocal regulation of Bcl-2 family proteins and activation of the intrinsic apoptotic pathway**

**Supplemental Figure Legends**

**Supplemental Figure 1. TPL synergistically interacts with ABT-199 to induce apoptosis in AML cell lines. (A-E)** p53 wild type (MV4-11, Molm13) and mutant/null human AML cells (KG-1α, U937, THP1) were treated as described in regular Figure 1E and 1F, after which the combination index (CI) was calculated at ED50, ED75 and ED90 using the CompuSyn software.

**Supplemental Figure 2. TPL is well tolerated at the dosed used in this study.** Non-immunocompromised mice (6-8 week-old male BALB/c mice) were given the same doses of TPL as described in Figure 3, after which blood cell account and liver function were monitored. WBC, white blood cells; RBC, red blood cells; HGB, hemoglobin; PLT, platelet; ALT, alanine transaminase; AST, aspartate transaminase; TP, total protein; ALB, albumin; TBIL, total bilirubin; ALP, alkaline phosphatase; BUN, blood urea nitrogen; CREA, creatinine.

**Supplemental Figure 3.** **12-hr exposure to TPL and ABT-199 alone or in combination induces modest apoptosis in AML cells.** MV4-11 and KG-1a cells were treated as described in Figure 5A and 5B for 12 hrs, after which flow cytometry was performed to monitor apoptosis after Annexin V and PI double staining.
