# Supplementary material for: Combining triptolide with ABT-199 is effective against acute myeloid leukemia through reciprocal regulation of Bcl-2 family proteins and activation of the intrinsic apoptotic pathway
Source: Cell Death Dis. 2020 Jul 22;11(7):555. doi: 10.1038/s41419-020-02762-w (PMC7376040; doi:10.1038/s41419-020-02762-w)
Supplement: Supplementary file 5 — Supplementary Table S1 [file 41419_2020_2762_MOESM5_ESM.docx]

**Combining Triptolide with ABT-199 is effective against acute myeloid leukemia through reciprocal regulation of Bcl-2 family proteins and activation of the intrinsic apoptotic pathway**

**Supplemental Table S1**

**Table S1.** Analysis of the interaction between ABT-199 and TPL using the Bliss Independence model in various AML cell lines

|  | The lowest conc.(nM) | | Interaction index | The highest conc. (nM) | | Interaction index |
| --- | --- | --- | --- | --- | --- | --- |
|  | ABT | TPL |  | ABT | TPL |  |
| MV4-11 | 1.25 | 1.25 | 0.311 | 5.0 | 5.0 | 0.599 |
| MOLM13 | 1.25 | 1.25 | 0.286 | 10 | 10 | 0.676 |
| KG1a CD34^+^/CD38^−^ | 10 | 1.25 | 0.136 | 160 | 20 | 0.565 |
| U937 | 250 | 2.5 | 0.217 | 1000 | 10 | 0.368 |
| THP-1 | 5.0 | 1.25 | 0.126 | 160 | 40 | 0.407 |
